# The association between HIV infection and pulmonary function in a rural African population

**DOI:** 10.1371/journal.pone.0210573

**Published:** 2019-01-15

**Authors:** Meri R. J. Varkila, Alinda G. Vos, Roos E. Barth, Hugo A. Tempelman, Walter L. J. Devillé, Roel A. Coutinho, Diederick E. Grobbee, Kerstin Klipstein-Grobusch

**Affiliations:** 1 Julius Global Health, Julius Center for Health Sciences and Primary Care, University Medical Center Utrecht, Utrecht University, Utrecht, the Netherlands; 2 Department of Internal Medicine & Infectious Diseases, University Medical Center Utrecht, Utrecht University, Utrecht, the Netherlands; 3 Wits Reproductive Health and HIV Institute, Faculty of Health Sciences, University of the Witwatersrand Johannesburg, Johannesburg, South Africa; 4 Ndlovu Care Group, Groblersdal, South Africa; 5 Julius Clinical Research, Academic Contract Research Organization, Zeist, the Netherlands; 6 Division of Epidemiology and Biostatistics, School of Public Health, Faculty of Health Sciences, University of the Witwatersrand, Johannesburg, South Africa; Massachusetts General Hospital, UNITED STATES

## Abstract

**Objectives:**

HIV infection has been associated with an impaired lung function in high-income countries, but the association between HIV infection and pulmonary function in Sub-Saharan Africa remains unclear. This study aims to investigate the relation between HIV infection and pulmonary function in a rural African population.

**Methods:**

A cross-sectional study was conducted among HIV-positive and HIV-negative adults in a rural area in South Africa, as part of the Ndlovu Cohort Study. A respiratory questionnaire and post-bronchodilator spirometry were performed. Multivariable regression analysis was used to investigate whether HIV was independently associated with a decrease in post-bronchodilator FEV_1_/FVC ratio considering age, sex, body mass index, respiratory risk factors and a history of a pulmonary infection (tuberculosis (TB) or a pneumonia). Possible mediation by a history of pulmonary infection was tested by removing this variable from the final model.

**Results:**

Two hundred and one consecutive participants were enrolled in the study in 2016, 84 (41.8%) were HIV-positive (82.1% on ART). The median age was 38 (IQR 29–51) years. Following multivariable analysis HIV was not significantly associated to a decline in post-bronchodilator FEV1/FVC ratio (β -0.017, p 0.18). However, upon removal of a history of a pulmonary infection from the final model HIV was significantly related to post-bronchodilator FEV_1_/FVC ratio, β -0.026, p 0.03.

**Conclusions:**

Pulmonary function is affected by HIV infection which most likely results from co-infection with TB or other pneumonia. Further research should focus on the influence of a pulmonary infection, most notably TB, on pulmonary function, especially as the incidence of TB is high in HIV infection.

## Introduction

Human Immunodeficiency virus (HIV) infection remains a major health problem in Sub-Saharan Africa (SSA). With nearly one in every 20 adults (4.9%) living with HIV in this part of the world, HIV/AIDS is the most important cause of morbidity and mortality in SSA [1,2].

Due to increased access to combination antiretroviral therapy (ART) the face of the epidemic is changing. Life expectancy of patients receiving ART is improving and long-term co-morbidities associated with HIV infection are becoming more relevant. Pulmonary emphysema and obstructive lung disease (OLD) are examples of frequently observed co-morbidities in HIV infection [3]. This intercedes with the existing burden of pulmonary diseases, with lower respiratory tract infections and tuberculosis ranking in the top five causes of years of life lost on the African continent [2].

OLD is more common in HIV-positive than in HIV-negative individuals, and HIV infection is independently associated with a decrease in lung function and diffusion capacity [4–10]. What role ART plays in the development of OLD is an unresolved issue. ART tapers inflammation, possibly reducing immunological damage on alveolar level, but a direct negative effect of ART on pulmonary function was also reported [4,11,12]. However, most data regarding OLD in HIV comes from high-income countries (HIC). Due to marked differences between the settings, such as sex distribution among HIV patients and occupational and environmental risk factors, these data cannot simply be applied to low- and middle-income countries (LMIC). [13,14]

Research into prevalence and pathogenesis of OLD in HIV in SSA is needed to guide medical management of HIV patients in low-resource settings and to reduce the burden of chronic pulmonary disease. This study sets out to investigate the relation between HIV infection and pulmonary function in a rural African population comparing HIV-positive and HIV-negative participants while taking pulmonary co-infection into account.

## Methods

### Study design and study population

Data was collected in a cross-sectional study, embedded in the Ndlovu Cohort Study (NCS). The NCS is a prospective study including approximately 2000 participants in rural South Africa (Limpopo province). The main aim is to investigate the epidemiology and pathogenesis of cardiovascular disease in the context of HIV infection. [15] Inclusion criteria for the NCS were age (≥18 years) and living in the proximity of the research site. All participants coming for a NCS baseline or follow-up visit in April and May 2016 were considered eligible for participation in this cross-sectional study. Exclusion criteria were myocardial infarction in the last month, surgery in the past six weeks, hypersensitivity to salbutamol or inability to undergo the procedure for any reason. The study was approved by the Human Ethics Research Committee of the University of Pretoria (Reference number 76/2016) and written informed consent was obtained from all subjects prior to study participation. The study was reported according to the STROBE guideline (S1 Appendix).

### Baseline data collection

Socio-demographic, lifestyle and anthropometric data, HIV-status and most recent laboratory results (CD4 count and HIV viral load) were collected from the NCS database. Data collection followed standardised procedures as previously described [15]. Additional data on a history of tuberculosis (TB), a history of a pneumonia, smoking, occupational and environmental exposure was collected using an adapted respiratory questionnaire (S2 Appendix) based on The British Medical Research Council Respiratory Questionnaire [16], World Health Survey [17], ATS-DLD-78-A [18], MRC breathlessness scale [19], and questions used in other publications [20–22].

### Spirometric measurements

Spirometry testing with pre- and post-bronchodilator measurements was performed using a hand-held CareFusion 2009 spirometer and Spida 5 software. Procedures and review of acceptability and repeatability were performed according to American Thoracic Society and European Respiratory Society guidelines [23]. At least three acceptable and repeatable blows were required where the subject performed the manoeuvre with a maximum inspiration, a good start, a smooth continuous exhalation, and maximal effort. In addition, the largest and second-largest values for both forced expiratory volume in 1 second (FEV_1_) and forced vital capacity (FVC) had to be within 150 mL of each other. In cases where acceptability and repeatability could not be achieved within three blows, the manoeuvre was repeated up to a maximum of eight times. After the initial measurements, a short-acting bronchodilator (salbutamol 100 μg, 4 doses) was administered using a spacer. Post-bronchodilator spirometry was repeated after a 15-minute waiting time using the same procedure as before. The respiratory parameters measured were: FEV_1_, FVC and FEV_1_/FVC ratio. The largest values for post-FEV_1_ and post-FVC were considered for analysis.

According to the Global Initiative for Chronic Obstructive Lung Disease (GOLD) OLD was defined as a FEV_1_/FVC-ratio below 0.70, post-bronchodilation [24]. As this definition can lead to underestimation of airflow limitation in young people and overestimation in older people, we chose to use a lower limit of normal (LLN) definition [25]. This was defined as a FEV_1_/FVC-ratio below the 5^th^ percentile of the predicted value. The Global Lung Initiative equation category “Afro-American” was used to define normal values [26]. Reversibility was defined as a post-bronchodilator increase in FEV_1_ of more than 12% and over 200 ml [27].

Spirometry tests that did not meet acceptability and repeatability criteria, and thus could not be evaluated for the presence of obstruction, were excluded from further analysis (n = 6).

### Statistical analysis

Continuous descriptive data was presented as means and standard deviations (SD) for normally distributed data and as medians and interquartile ranges (IQR) for non-normally distributed data. Categorical descriptive data was presented as frequencies and percentages. Selection bias was evaluated by comparing demographics of those who were enrolled in this sub-study to the demographics of the total NCS. The comparison included age, sex, HIV status, education level, marital status and body mass index (BMI). We used a t-test for continuous outcomes and a Chi square test for dichotomous outcomes.

Post-bronchodilator FEV_1_ and FVC outcomes, post-bronchodilation FEV_1_/FVC-ratio and the prevalence of OLD were compared between HIV-positive and HIV-negative individuals.

The association of HIV and FEV_1_/FVC ratio was assessed using the fixed post- bronchodilator FEV_1_/FVC ratio. We decided not to use the percentage of predicted FEV_1_/FVC generated by the GLI equation category “Afro-American”, as it is not certain if these values reflect the normal distribution in a Black African population. The relation between HIV and post-bronchodilator FEV_1_/FVC ratio was tested in a linear regression analysis. The following baseline variables were considered as confounders and tested in univariable analysis: age, gender, BMI, education, employment, current or ever smoking cigarettes or cigars, current smoking of marijuana, indoor smoking in childhood, a history of TB or pneumonia, use of wood fire outdoors and occupational exposure (mining work, having worked in a dusty job for more than a year or having working with chemicals and fumes for more than one year). All variables with p-value <0.2 in univariable analysis as well as age, sex and BMI (regardless of the p-value) were entered in a multivariable analysis. Second, we assessed mediation by a history of tuberculosis (TB) on the relation between HIV and post-bronchodilator FEV_1_/FVC ratio by removing TB from the final model. Analysis was performed using SPSS software (IBM Corp. Released 2016. IBM SPSS Statistics for Windows, Version 24.0. Armonk, NY: IBM Corp.).

## Results

**Fig 1:**
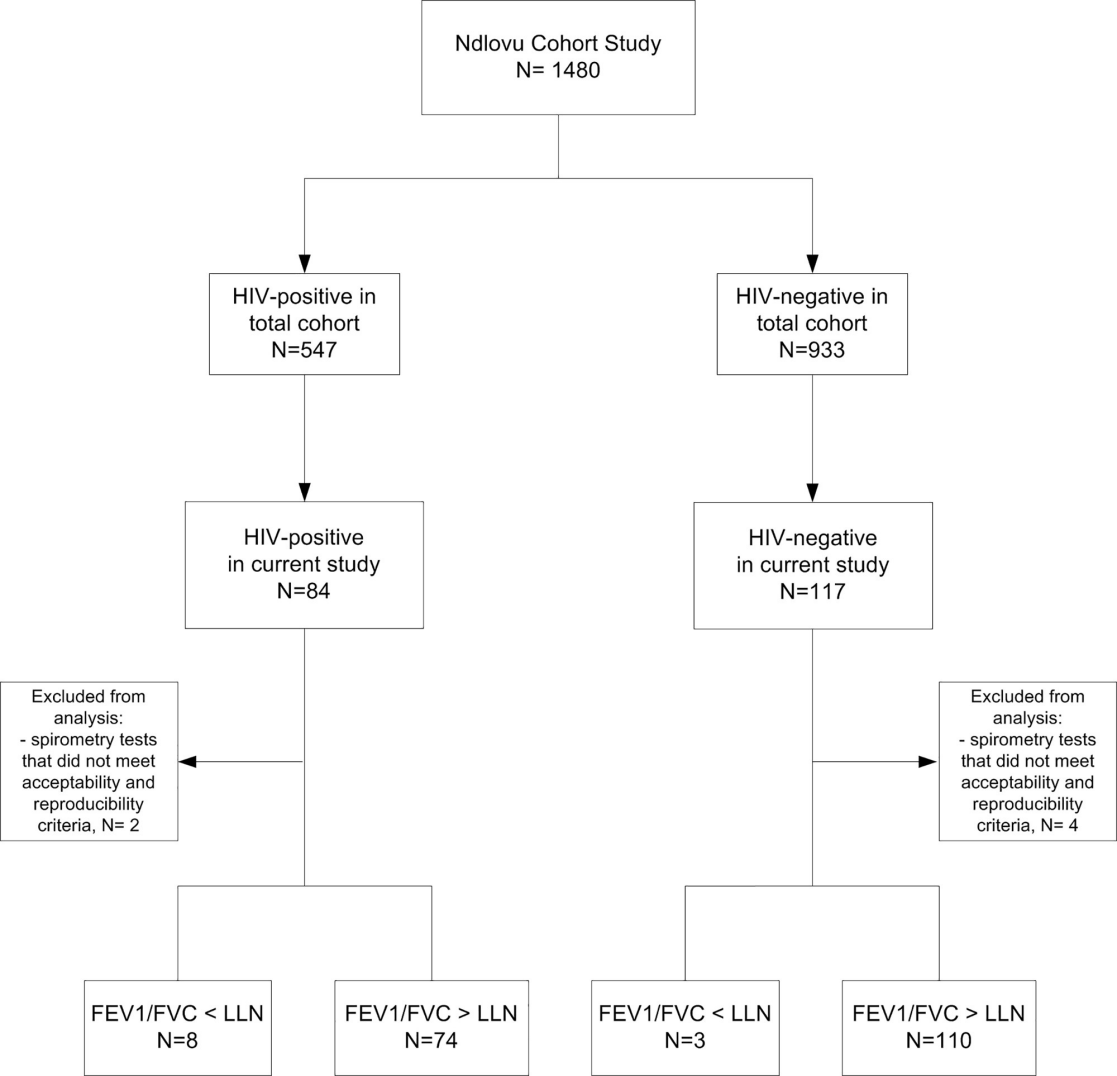
Subject recruitment.

Two hundred and one participants (50% men) were enrolled in the study, 84 (42%) of whom were HIV-positive (Fig 1, Table 1). The age of participants in this sub-study was the only demographic factor that differed compared to the demographics of the participants in the NCS (the age in this sub-study was on average 2 years higher than the age in the NCS, p = 0.03) (S1 Table). The proportion of men was higher in the HIV-negative group (65%) as compared to the HIV-positive group (30%). HIV-positive participants were slightly older than HIV-negative participants.

**Table 1:** Baseline characteristics of study population.

	HIV-positive (n = 84)	HIV-negative (n = 117)	*P* value
**Demographic characteristics**			
Men	25 (29.8%)	76 (65.0%)	<0.01
Age	42.4 (10.4)	39.0 (14.8)	0.08
Education level			0.37
None or Primary	21 (25.0%)	30 (25.6%)	
Secondary or higher	63 (75.0%)	87 (74.4%)	
Employment status			0.02
Unemployed	41 (48.8%)	55 (47.0%)	
Employed	34 (40.5%)	34 (29.1%)	
Retired	9 (10.7%)	17 (14.5%)	
Student	0 (0.0%)	11 (9.4%)	
Tobacco smoking			<0.01
Never smoking	57 (67.9%)	50 (42.7%)	
Current smoking	15 (17.9%)	46 (39.3%)	
Former smoking	12 (14.3%)	21 (17.9%)	
No. cigarettes smoked daily*	8.8 (9.3)	6.3 (3.8)	0.40
Marijuana smoking	10 (11.9%)	27 (23.1%)	0.04
Cooking fuel			0.83
Gas	6(7.1%)	5 (4.3%)	
Electricity	73 (86.9%)	104 (88.9%)	
Paraffin	2 (2.4%)	3 (2.6%)	
Kerosene	0 (0.0%)	1 (0.9%)	
Solid fuels	3 (3.6%)	4 (3.4%)	
Use of fuel of heating (n = 77)			0.28
Gas	0 (0.0%)	3 (6.7%)	
Electricity	31 (96.9%)	39 (86.7%)	
Kerosene	0 (0.0%)	2 (4.4%)	
Solid fuels	1 (3.1%)	1 (2.2%)	
Occupational exposure			
Mining work	5 (6.0%)	15 (12.8%)	0.11
Dusty job ≥1 year	12 (14.3%)	26 (22.2%)	0.16
Chemicals or fumes ≥ 1 year	5 (6.0%)	9 (7.7%)	0.63
Medical history			
Pneumonia	8 (9.5%)	2 (1.7%)	0.01
Pulmonary TB	29 (34.5%)	6 (5.1%)	<0.01
**HIV-related characteristics**			
Years since diagnosis, mean (SD)	5.3 (4.5)	-	
On ART	69 (82.1%)		
First line	68 (98.6%)	-	
Years since start ART, median (IQR)	5.0 (2.0–8.0)	-	
CD4 count cells/mm3 (n = 83)			
<200	13 (15.7%)	-	
200–350	11 (13.3%)	-	
>350	59 (71.1%)	-	
Viral load cp/ml (n = 78)			
<50	54 (69.2%)	-	
51–1000	7 (9.0%)	-	
>1000	17 (21.8%)	-	

IQR, interquartile range, TB, tuberculosis.

Data are in n (%) or mean (SD) unless otherwise specified.

* Data for current smokers.

Differences in socioeconomic, environmental, and occupational factors between the HIV-positive group and HIV-negative controls were minor. Current smoking was reported by 61 participants; 46 (75%) were HIV negative. Almost all smokers were males (n = 57, 93%). Occupational exposure was generally low and did not differ by HIV status. There were marked differences between men and women: 19% of all men reported having worked in a mine versus 1% of women, 32% of men had worked in a dusty job for more than one year versus 6% of women and 13% of men had worked in a job with exposure to chemical fumes or gases for more than one year compared to 1% of women. A history of pulmonary tuberculosis or pneumonia was reported significantly more often by HIV-positive than by HIV-negative participants (Table 1).

Acceptable spirometry test results could be obtained in 195 (97%) participants. Post-FEV_1_ and -FEV_1_/FVC values were significantly lower in the HIV-positive group than the HIV-negative group (Table 2). The prevalence of OLD, according to the LLN, was 10% (n = 8) in HIV-positive participants and 3% (n = 3) in HIV-negative participants (p = 0.06).

**Table 2:** Post-bronchodilator spirometry results.

	All (n = 195)	HIV-positive (n = 82)	HIV-negative (n = 113)	*P* value
FEV_1_ (L)	2.94 (0.79)	2.62 (0.66)	3.17 (0.80)	<0.01
FEV_1_% predicted*	101.0 (16.3)	96.4 (17.7)	104.0 (14.5)	<0.01
FVC (L)	3.51 (0.81)	3.22 ((0.71)	3.73 (0.82)	<0.01
FVC% predicted*	99.8 (14.5)	97.4 (15.2)	101.4(13.8)	0.05
FEV_1_/FVC-ratio	0.84 (0.09)	0.82 (0.10)	0.85 (0.08)	0.03
FEV_1_/FVC-ratio predicted*	101.1 (9.9)	99.3 (12.2)	102.3 (7.5)	0.04
FEV_1_ < 80% predicted*	16 (8.2%)	11 (13.4%)	5 (4.4%)	0.02
FEV_1_/FVC < 0.70	9 (4.6%)	6 (7.3%)	3 (2.7%)	0.17
FEV_1_/FVC < LLN*	11 (5.6%)	8 (9.8%)	3 (2.7%)	0.06

FEV_1_ forced expiratory volume in 1 s; FVC, forced vital capacity; LLN, lower limit of normal

Data in mean (SE) or n(%).

* Based on GLI reference equations “Afro-American”

HIV was independently associated with a decrease in post- bronchodilator FEV_1_/FVC ratio following univariable regression analysis, β -0.028, p = 0.03.

Following multivariable regression analysis HIV was not related to post-bronchodilator FEV1/FVC ratio anymore (Table 3). However, when a history of TB or pneumonia was removed from the model the relation between HIV and post-bronchodilator FEV_1_/FVC ratio became significant again, indicating that the relation between HIV and post-bronchodilator FEV_1_/FVC ratio was mediated by TB (Table 3).The small number of HIV-positive participants not on ART (n = 15) did not allow us to evaluate the effect of ART on the post- bronchodilator FEV_1_/FVC ratio.

**Table 3:** Uni- and multivariable analysis and mediation analysis.

Post FEV1/FVC	Univariable Unstandardized β coefficient (95% CI)	*p*	Multivariable Unstandardized β coefficient (95% CI)	*p*	Mediation Unstandardized β coefficient (95% CI)	*p*
HIV	-0.028 (-0.054–-0.003)	0.030	-0.017 (-0.043–0.008)	0.177	-0.026 (-0.050–-0.003)	0.030
Age	-0.004 (-0.004–-0.003)	<0.001	-0.003 (-0.004–-0.002)	<0.001	-0.003 (-0.004–-0.002)	<0.001
Female gender	0.012 (-0.014–0.037)	0.357	0.002 (-0.027–0.031)	0.878	0.004 (-0.025–0.033)	0.778
Body mass index (kg/m^2^)	0.000 (-0.002–0.003)	0.675	0.001 (-0.001–0.003)	0.315	0.001 (-0.001–0.003)	0.281
History of tuberculosis or a pneumonia	-0.047 (-0.078–-0.016)	0.003	-0.026 (-0.055–0.003)	0.078		
Education, secondary or higher	0.025 (0.013–0.037)	<0.001	0.001 (-0.012–0.014)	0.934	0.001 (-0.012–0.014)	0.885
Employment^1^	0.009 (-0.018–0.036)	0.513				
Indoor smoking in childhood	-0.027 (-0.056–0.003)	0.074	-0.020 (-0.046–0.005)	0.116	-0.019 (-0.045–0.006)	0.137
Current or ever smoking	-0.027 (-0.052–-0.001)	0.039	-0.007 (-0.034–0.020)	0.620	-0.005 (0.032–0.022)	0.732
Current smoking marijuana	0.019 (-0.025–0.063)	0.402				
Exposure to wood fire outdoors	0.012 (-0.020–0.044)	0.467				
Occupational exposure^2^	-0.039 (-0.066–-0.012)	0.005	-0.026 (-0.053–0.001)	0.062	-0.027 (-0.054–0.000)	0.048

CI; confidence interval.

^1^Employed versus no employment, retired or student.

^2^Defined as any of the following: mining work, exposure to chemicals or fumes at work or worked in a dusty environment, all for more than a year.

## Discussion

In this African cohort the prevalence of OLD was 10% in HIV-positive participants compared to 3% in the HIV-negative group. HIV infection was not related to the post- bronchodilator FEV_1_/FVC ratio in multivariable analysis. However, when a history of a pulmonary infection was removed from the final model HIV became significantly associated with a decline in post-bronchodilator FEV_1_/FVC ratio. This indicates that a history of a pulmonary infection mediates the relation between HIV infection and a decline in pulmonary function.

Our results on the prevalence of OLD are roughly in line with the estimated worldwide prevalence of OLD of 11% [28] and 10.5% in people living with HIV [29]. Studies conducted amongst general populations in African countries reported a prevalence ranging from 4.1% to 24.8% [30], and 6.0% to 22.2% in HIV-positive populations[29,31–33]. Although not statistically significant in our cohort, the higher prevalence of OLD in HIV-positive participants is also in line with data from both HIC and LMIC. [5,6,9, 31]

Two recent studies conducted in HIV-positive cohorts found that immune defects as measured by a lower CD4 T cell count may be associated with a decline in FEV_1_ and FVC [34] and COPD [35]. In a large, international randomized controlled trial, the timing of ART initiation had no effect on rate of lung function decline in ART-naïve individuals with CD4 T cell counts of more than 500 per μL [36]. These findings support the newly updated WHO recommendations that all patients should be treated with ART regardless of their CD4 T cell counts [37]. Data from SSA countries regarding the role of immunomodulation and HIV and the effect of ART on OLD still remains limited.

In TB-endemic regions, such as SSA, the epidemiology of HIV infection and TB tend to be highly intertwined. Up to 65% of South Africans with TB also tested positive for HIV [38], and vice versa, globally approximately 30% of HIV-positive persons are estimated to have (usually latent) *M*. *tuberculosis* infection [39]. The risk of active TB doubles within the first year of HIV infection [40] and the life-time risk of active TB for HIV/TB co-infected persons in Africa has been estimated to be around 30–40% [41]. These figures highlight the relationship between HIV infection and pulmonary coinfection in SSA, yet few studies have taken the possible contribution of TB/pneumonia into account when looking at HIV and changes in lung function. Recent work investigating HIV infection, TB and COPD in a Ugandan cohort found a higher prevalence of COPD in HIV-positive individuals with a prior history of TB [33]. These findings are in agreement with our results and suggest decreased lung function in HIV infection in a SSA setting could be attributed to pulmonary co-infection rather than HIV infection.

Whereas associations between prior history of pneumonia and OLD have been established [42–44], the pathophysiological mechanisms behind airflow obstruction as a result of pulmonary infection are still incompletely understood. Postulated pathways include the development of bronchiectasis and bronchial stenosis due to aberrant lung tissue remodelling after inflammatory injury [44], as well as chronic inflammation caused by dysregulated macrophages [45]. The pathophysiological interactions of chronic inflammation in HIV infection and lung injury caused by pulmonary infections present interesting topics for future research.

Our analysis also showed that age was robustly associated with a decline in post-bronchodilator FEV_1_/FVC ratio. This was to be expected as pulmonary function decreases with age [46–49]. We also noted that the widely studied OLD-risk factor smoking was not associated with a decline in the post-FEV_1_/FVC ratio following multivariable analysis (either passive smoking in childhood, or former and current smoking versus never smoking). This may be due to the sample size, as well as different smoking habits between high income settings and low income settings [50, 51]. In our study smoking habits were often described as irregular and highly dependent on fluctuations in participants’ finances (average consumption of 7 cigarettes per day).

Some limitations of our study need to be mentioned. First, as a result of the small sample size we could not investigate the clinically relevant outcome OLD. However, we do not expect that HIV would be associated with OLD, as we found no relation with post-bronchodilator FEV_1_/FVC when a history of a pulmonary infection was considered. Due to the relatively low number of study participants we were also unable to explore the role of other HIV-related factors, such as ART, CD4 T cell counts and viral loads. Second, as our data collection was constrained to one time-point, studying changes in lung function in HIV infection was beyond the scope of this study. Third, the cross-sectional design limits causal inference. Fourth, participants in this analysis were slightly older than participants in the NCS. However, the other demographic factors did not differ, so it is unlikely that this sub-study is subjected to selection bias. The older age is also unlikely to influence the generalizability of our findings to all cohort participants as there is no reason to assume that the influence of HIV on pulmonary function would change with a small increase in age. Finally, for some of the data we relied on self-reporting, which may have subjected some of our measured parameters to recall or reporting bias.

Strengths to be mentioned are the comparison between HIV-positive and HIV-negative individuals, the rural setting and the extensive evaluation of pulmonary risk factors like smoking in childhood and occupational exposure. As a result, our study contributes to the current understanding on the influence of HIV on post-bronchodilator FEV_1_/FVC ratio and it highlights the influence of a history of a pulmonary infection on pulmonary function in a rural SSA setting

In conclusion, HIV infection is associated with a decline in pulmonary function measured with post-bronchodilator FEV_1_/FVC ratio, but this effect seems to be mediated by a history of a pulmonary infection, notably TB. TB is still highly prevalent, especially in the HIV-positive population and may pose a significant burden of pulmonary disease in these patients. We would therefore recommend to investigate the influence of TB on pulmonary function, considering HIV infection, in a larger cohort using the clinically more relevant outcome ‘OLD’. In the meantime, the regular contact of HIV-positive participants with the health care system should be used to direct clinical care towards prevention and early treatment of TB infections.

## Supporting information

S1 Appendix STROBE statement. (DOC)

S2 Appendix Questionnaire. (PDF)

S1 Table Comparison of demographics between the current study and the total cohort Ndlovu Cohort Study). (DOCX)
