# Supplementary material for: The association between HIV infection and pulmonary function in a rural African population
Source: PLoS One. 2019 Jan 15;14(1):e0210573. doi: 10.1371/journal.pone.0210573 (PMC6333365; doi:10.1371/journal.pone.0210573)
Supplement: S2 Appendix — (PDF) [file pone.0210573.s002.pdf]

**Baseline questionnaire about HIV characteristics, demographics, lifestyle and medication use**<br><br>**Breath study**	**Study number:**
	**Age:**
	**Date of visit:**
	**Fieldworker:**

This questionnaire will take about 15 minutes. The questions are meant to get insight into pulmonary complaints and risk factors for lung diseases. Because a broad range of men and women are participating in the study, some questions in this interview may not apply to you. However we ask the same questions to all participants. Your answers are completely confidential. Your responses are linked only to confidential study number, not to your name. In case of any questions or troubles do not hesitate to ask the investigator.

The next questions are about pulmonary complaints and risk factors for pulmonary diseases. *Please thick '**Yes**' or '**No**' whenever possible.* If you do not know an answer, or if a question does not apply to you, thick 'unknown'.

**Cough**	**Yes**	**No**	**Unknown**
1. Do you cough several times most day?	☐	☐ →go to 5	☐
If Yes to 1			
2. When do you cough?	☐ I wake up with cough<br>☐ In the morning<br>☐ During the day<br>☐ During the night		
3. Is it a chronic cough? (*lasting for more than 2 consecutive months)*	☐	☐	☐
4. For how long have you been coughing?			………..years
**Sputum**	☐	☐	☐
5. Do you bring up phlegm or mucus on most days?	☐	☐ →go to Q8	☐ →go to Q8
If Yes to 5			
6. When do you bring up phlegm or mucus?	☐ first thing in the morning<br>☐ during the day		
7. Do you have a chronic phlegm? (*lasting for more than 2 consecutive months)*	☐	☐	☐

MRC Breathlessness Scale

If you are disabled from walking by any condition other than heart or lung disease, omit question 20 and enter '1' here

8. Which of the following statements best describes your situation?

☐1 Not troubled by breathlessness except on strenuous exercise
☐2 Short of breath when hurrying on the level or walking up a slight hill
☐3 Walks slower than most people on the level, stops after a mile or so, or stops after 15 minutes walking at own pace
☐4 Stops for breath after walking about 100 yards or after walking a few minutes in level ground
☐5 Too breathless to leave house, or breathless when undressing

Wheezing

9. Have you had attacks of wheezing or whistling in your chest at any time in the last 12 months?	☐	☐ →go to Q14	☐ →go to Q14
10. Have you been at all breathless when wheezing was present?	☐	☐	☐
11. Is/was your breathing absolutely normal between attacks?	☐	☐	☐
12. Do you wake up with wheezing?	☐	☐	☐
13. For how long do you wheeze or have any whistling on the chest?	……….years		

Exacerbations

14. Did you have periods of increased breathing difficulty with increased cough with or without sputum during the last 12 months?	☐	☐ →go to Q16	☐ →go to Q16
If Yes to 14			
15. How many times did you have such period during the last 12 months?	……….times		

Past illnesses

Have you ever had, or been told that you have had:

	Yes	No	Unknown
16a. An injury or operation affecting your chest	☐	☐	☐
16b. Heart trouble	☐	☐	☐

16c. Pneumonia	☐	☐	☐
16d. Bronchial asthma	☐	☐	☐
16e. Hay fever	☐	☐	☐
16f. Other chest trouble	☐	☐	☐
If yes to one of the above mentioned questions (16a-16f)			
16g. Did/do you receive treatment for this/these condition(s)?	☐	☐	☐
17. Did you ever have tuberculosis?	☐ Yes, I have active TB now<br>☐ Yes, I had TB in the past but was treated and I am cured now<br>☐ Yes, I had TB in the past but I could not complete treatment<br>☐ No, I never had TB<br>☐ I don't know		
If yes to 17			
18. In which year where you diagnosed with TB?	year		
19. For how long did you take or do you have to take treatment?	☐ 6 months<br>☐ 9 months<br>☐ other..		
**Occupational history** (ATS-DLD-78-A)			
	**Yes**	**No**	**Unknown**
20. Have you ever worked for a year or more in the mining industry?	☐	☐	☐
If yes to 20			
21. For how long did you work in the mining industry?	Total years worked:		
	**Yes**	**No**	**Unknown**
22. Have you ever worked for a year or more in a dusty job (except the mining industry)?	☐	☐	☐
If yes to 22			
23. For how long did you work in this dusty job?	Total years worked:		
	**Mild**	**Moderate**	**Severe**
24. Was dust exposure mild, moderate or severe? *(please indicate the category)*	☐	☐	☐
	**Yes**	**No**	**unknown**
25. Have you ever been exposed to gas or chemical fumes in your work?	☐	☐	☐
If yes to 25			

26. Please specify job/industry:	Total years worked:		
	**Mild**	**Moderate**	**Severe**
27. Was gas or chemical fumes exposure mild, moderate or severe? *(please indicate the category)*	☐	☐	☐
28. What has been your usual occupation or job / the one you have worked at the longest?			
29. Number of years employed in this occupation			years
30. What is your current occupation?			

**Indoor air pollution** (WHO World health survey 2002)		
31. What type of fuel does your household mainly use for cooking?	Gas	☐
	electricity	☐
	Paraffin	☐
	Kerosene	☐
	Coal	☐
	Charcoal	☐
	Wood	☐
	Agriculture/crop	☐
	Animal dung	☐
	Shrubs/grass	☐
	Other:	☐
32. What type of cooking stove is used in your house?	Open fire or stove without chimney or hood	☐
	Open fire or stove with chimney or hood	☐
	Closed stove with chimney	☐
	Other	☐
33. What type of house do you have?	Flat	☐
	Self-contain / house	☐
	One room	☐
	Passage	☐
34. Where is cooking usually done?	In a room used for living or sleeping	☐
	In a separate room used as kitchen	☐
	In a separate building used as	☐

	kitchen	
	Outdoors	☐
35. Do you heat your house when it is cold?	Yes ☐	No ☐ →go to Q40
36. What type of fuel does your household mainly use for heating?	Gas	☐
	Electricity	☐
	Paraffin	☐
	Kerosene	☐
	Coal	☐
	Charcoal	☐
	Wood	☐
	Agriculture/crop	☐
	Animal dung	☐
	Shrubs/grass	☐
	Other:	☐
37. What type of heating stove is used in your house?	Open fire or stove without chimney or hood	☐
	Open fire or stove with chimney or hood	☐
	Closed stove with chimney	☐
	Other	☐
38. How much time do you on average spent in the cooking place per day?	More than 6 hours	☐
	4-6 hours	☐
	1-3 hours	☐
	Less than 1 hour	☐
39. How much time do you on average spent close to a fire per day?	More than 6 hours	☐
	4-6 hours	☐
	1-3 hours	☐
	Less than 1 hour	☐
40. Does anybody in your household smoke inside the house?	Yes ☐	No ☐
**Family history of pulmonary diseases *(ATS-DLD-78a)***		
41. Did either of your natural parents have a chronic lung condition?	Yes ☐	No ☐
If yes to 41		
42. Please specify which disease is was:		

**Exposure in the past**			
	**Yes**	**No**	**unknown**
43. Did anybody in your household smoke in	☐	☐	☐

childhood?			
44. Did anybody in your household smoke insight the house in childhood?	☐	☐	☐
45. Was cooking done indoors or was there a fire place insight the house in childhood?	☐	☐	☐
**General**			
	**Yes**	**No**	**unknown**
46. Did you ever use intravenous drugs?	☐	☐ →End of questionnaire	☐ →End of questionnaire
47. Did you use intravenous drugs in the past 3 months?	☐	☐ →End of questionnaire	☐ →End of questionnaire
48. How often do you use intravenous drugs?	....times per month/week (please indicate which)		

End of questionnaire. Thanks for your participation!
