# Supplementary material for: The association between HIV infection and pulmonary function in a rural African population
Source: PLoS One. 2019 Jan 15;14(1):e0210573. doi: 10.1371/journal.pone.0210573 (PMC6333365; doi:10.1371/journal.pone.0210573)
Supplement: S1 Table — (DOCX) [file pone.0210573.s003.docx]

|  | **Current study**  n=201 | **Total cohort**  n=1927 | ***p*** |
| --- | --- | --- | --- |
| Age (years) | 40.9 (13.2) | 38.7 (12.8) | 0.025 |
| Female gender | 100 (49.8%) | 1056 (54.8%) | 0.171 |
| HIV-positive | 84 (41.8%) | 887 (46.0%) | 0.251 |
| More than primary education | 150 (74.6%) | 1467 (76.1%) | 0.635 |
| Married or a long-term relation | 129 (64.2%) | 1123 (58.3%) | 0.106 |
| Body mass index (kg/m^2^) | 24.0 (5.9) | 24.1 (6.1) | 0.752 |
| Values in mean with standard deviation and count with percentage. N, number | | | |

**Supplementary table** Comparison of demographics between the current study and the total cohort (Ndlovu Cohort Study)
